# Mitochondrial Succinate Metabolism and Reactive Oxygen Species Are Important but Not Essential for Eliciting Carotid Body and Ventilatory Responses to Hypoxia in the Rat

**DOI:** 10.3390/antiox10060840

**Published:** 2021-05-25

**Authors:** Agnieszka Swiderska, Andrew M. Coney, Abdulaziz A. Alzahrani, Hayyaf S. Aldossary, Nikolaos Batis, Clare J. Ray, Prem Kumar, Andrew P. Holmes

**Affiliations:** 1 Institute of Clinical Sciences, University of Birmingham, Edgbaston, Birmingham B15 2TT, UK; agnieszka.swiderska@postgrad.manchester.ac.uk (A.S.); a.m.coney@bham.ac.uk (A.M.C.); AAA717@student.bham.ac.uk (A.A.A.); HXA807@student.bham.ac.uk (H.S.A.); c.j.ray@bham.ac.uk (C.J.R.); p.kumar@bham.ac.uk (P.K.); 2 Manchester Academic Health Sciences Centre, Unit of Cardiac Physiology, 3.26 Core Technology Facility, Institute of Cardiovascular Sciences, The University of Manchester, 46 Grafton Street, Manchester M13 9NT, UK; 3 Respiratory Care Department, Faculty of Applied Medical Sciences, Umm Al-Qura University, Makkah 24381, Saudi Arabia; 4 Basic Medical Sciences, College of Medicine, King Saud bin Abdulaziz University for Health Sciences, Riyadh 11481, Saudi Arabia; 5 Institute of Cancer and Genomic Sciences, University of Birmingham, Edgbaston, Birmingham B15 2TT, UK; N.Batis@bham.ac.uk; 6 Institute of Cardiovascular Sciences, University of Birmingham, Edgbaston, Birmingham B15 2TT, UK

**Keywords:** carotid body, hypoxia, succinate, mitochondrial reactive oxygen species, succinate dehydrogenase, hypoxic ventilatory response

## Abstract

Reflex increases in breathing in response to acute hypoxia are dependent on activation of the carotid body (CB)—A specialised peripheral chemoreceptor. Central to CB O_2_-sensing is their unique mitochondria but the link between mitochondrial inhibition and cellular stimulation is unresolved. The objective of this study was to evaluate if ex vivo intact CB nerve activity and in vivo whole body ventilatory responses to hypoxia were modified by alterations in succinate metabolism and mitochondrial ROS (mitoROS) generation in the rat. Application of diethyl succinate (DESucc) caused concentration-dependent increases in chemoafferent frequency measuring approximately 10–30% of that induced by severe hypoxia. Inhibition of mitochondrial succinate metabolism by dimethyl malonate (DMM) evoked basal excitation and attenuated the rise in chemoafferent activity in hypoxia. However, approximately 50% of the response to hypoxia was preserved. MitoTEMPO (MitoT) and 10-(6′-plastoquinonyl) decyltriphenylphosphonium (SKQ1) (mitochondrial antioxidants) decreased chemoafferent activity in hypoxia by approximately 20–50%. In awake animals, MitoT and SKQ1 attenuated the rise in respiratory frequency during hypoxia, and SKQ1 also significantly blunted the overall hypoxic ventilatory response (HVR) by approximately 20%. Thus, whilst the data support a role for succinate and mitoROS in CB and whole body O_2_-sensing in the rat, they are not the sole mediators. Treatment of the CB with mitochondrial selective antioxidants may offer a new approach for treating CB-related cardiovascular–respiratory disorders.

## 1. Introduction

The ability for humans to sense and respond to a fall in blood oxygen (hypoxia/hypoxaemia) has never been so apparent as in the current COVID-19 pandemic, in which millions of people have experienced this life-threatening stressor [1]. When challenged by hypoxia, the carotid body (CB) is the major peripheral chemoreceptor that detects this stimulus within seconds [2,3]. In contrast to almost all other cell types, the CB type I cell has an extraordinarily high sensitivity to O_2_, with its activity increasing exponentially from mild levels of hypoxia [4]. Upon stimulation, the CB activates numerous critical protective reflexes including hyperventilation, tachycardia, systemic vasoconstriction, and adrenaline release [5,6]. These reflexes are essential to preserve enough O_2_ delivery to the brain and vital organs, helping to support survival.

What remains controversial is the precise mechanism by which the CB senses hypoxia, with many different mechanisms being proposed [7]. One of the most longstanding hypotheses is that during hypoxia, CB mitochondrial electron transport is inhibited [8,9]. Importantly, the CBs express unique mitochondria which have a much lower O_2_ affinity in contrast with other cell types [8,9,10,11]. Functional experiments have shown that mitochondrial inhibition in CB type I cells starts to occur as the PO_2_ falls below a threshold of between 40–60 mmHg, i.e., considerably above the normal value of less than 5 mmHg observed in other cells [9]. As such, CB mitochondrial inhibition, activation of the chemotransduction cascade, and initiation of protective reflexes can be achieved in response to relatively small falls in blood PO_2_ from normoxic values and before the metabolism or function of other cells starts to be impaired [11].

A key consideration is the link between mitochondrial inhibition and activation of the downstream chemotransduction cascade [11,12]. Suggested mechanisms include a rise in [lactate]_i_ [13], a fall in [MgATP]_i_ [14,15], and/or stimulation of AMP-activated protein kinase (AMPK) [16]. A recent hypothesis is that during hypoxia, an elevation in mitochondrial reactive oxygen species (mitoROS) generation at complex I is sufficient to cause membrane K^+^ channel closure and chemostimulation [17,18]. Mice lacking the complex I *ndufs2* gene do not display an increase in respiratory frequency when subjected to hypoxia [17]. CB type I cells isolated from *ndufs2* deficient mice do not exhibit rises in either mitoROS or intracellular Ca^2+^ in response to hypoxia [17,18]. Importantly, the elevation in mitoROS is proposed to be dependent on a rise in succinate metabolism at complex II, reverse electron transport (RET), and the oxidation of ubiquinol (QH_2_) to ubiquinone (Q) at complex I [18,19]. It is currently unclear if selective pharmacological targeting of mitochondria with antioxidants can abolish or dampen the CB chemoafferent or whole animal response to hypoxia. Information of this type should underpin development of treatments for CB hyperactivity, an emerging driver of neurogenic hypertension [20,21].

Countering the idea that complex I-derived mitoROS are essential for CB O_2_ sensing is the finding that type I cell hypoxic sensitivity can be retained in the presence of rotenone (complex I inhibitor) by feeding electrons directly to cytochrome c [22]. Furthermore, CB type I cells isolated from mice with heterozygous deletion of the mitochondrial complex II gene *SDHD* (encoding succinate dehydrogenase (SDH) subunit D) display the same neurosecretory response to hypoxia as those obtained from wildtype littermates, raising questions about the importance of succinate in hypoxic chemotransduction [23]. No study has directly examined the role of succinate metabolism and mitoROS generation in mediating CB or ventilatory O_2_ sensitivity in the rat.

The aim of the current study was to evaluate if ex vivo CB chemoafferent activity and in vivo ventilatory responses to hypoxia could be modified by alterations in succinate metabolism and mitoROS signalling in the rat.

## 2. Materials and Methods

### 2.1. Ethical Approval

All procedures were performed in accordance with UK Animals (Scientific Procedures) Act 1986 and approved by the UK Home Office (PPL number PF4C074AD) and by the Animal Welfare and Ethical Review Body (AWERB) at the University of Birmingham. Adult male Wistar rats (*n* = 98, 5–10 weeks, 120–390 g) were purchased from Charles River, UK. Animals were housed in individually ventilated cages (*n* = 2–4 per cage) under standard conditions: 12:12 h light:dark cycle (lights on at 0700), 22 °C and 55% humidity. Food and water were available ad libitum. Animals were killed humanely by either exposure to carbon dioxide gas in a rising concentration or by dislocation of the neck (following removal of CBs under terminal non recovery anaesthesia).

### 2.2. Recordings of CB Chemoafferent Nerve Activity

CBs were isolated from adult male Wistar rats under deep non-recovery terminal inhalation anaesthesia (3–5% isoflurane in O_2_, 1.5 L min^−1^) as previously described [24,25]. Depth of anaesthesia was monitored during the procedure by absence of a hind limb flexor withdrawal reflex and breathing frequency.

Intact carotid bifurcations containing the carotid sinus nerve (CSN) and CB were removed and animals immediately killed by cervical dislocation. The tissue was transferred to a recording chamber (volume of approximately 0.2 mL) with a Sylgard 184 base (Dow Corning, Midland, MI, USA) and continuously superfused with a bicarbonate buffered Krebs solution containing, in mM: 119 NaCl, 4.5 KCl, 1.2 NaH_2_PO_4_, 1.2 MgSO_4_.7H_2_O, 25 NaHCO_3_, 2.4 CaCl_2_, and 11 D-glucose, 37 °C, pH 7.4, equilibrated with 95% O_2_, 5% CO_2_. The CSN was identified, dissected-free and surrounding connective tissue removed. To aid with extracellular recording [26,27], the tissue was partially digested in a Krebs solution containing 0.075 mg mL^−1^ collagenase type II and 0.0025 mg mL^−1^ dispase type I (Sigma-Aldrich, Gillingham, UK), for 20–30 min.

Extracellular action potential recordings of single and few-fibre units were recorded from the cut end of the CSN using borosilicate glass pipettes as described [28,29]. Acquisition and analysis were performed using Spike2 (version 7.12) software (Cambridge Electronic Design, Cambridge, UK). Raw chemoafferent voltage was amplified x5000 and sampled at 15 kHz. Single units were used for frequency analysis. These were discriminated initially by thresholding and subsequently by comparison of specific AP waveform parameters such as amplitude, 50% repolarisation time, and time to peak hyperpolarisation.

Experiments were performed at 37 °C and superfusate PO_2_ was continuously measured (100 Hz) using an O_2_ electrode (ISO_2_) and O_2_ meter (OXELP; World Precision Instruments, Hitchin, UK). Basal activity was measured at ca. 300 mmHg and hypoxic responses were induced by a slow ramp down to ca. 100 mmHg before rapid reversal into hyperoxia (95% O_2_, 5% CO_2_). Single fibre frequency was plotted against the superfusate PO_2_ and data fitted to an exponential curve with offset:

y = a + b × Exp(^−cx^) (1)

where *y* is the discharge frequency (Hz), *x* is the superfusate PO_2_ (mmHg), *a* is the discharge frequency as the PO_2_ tends to infinity (offset), *b* is the theoretical frequency when the PO_2_ is 0 mmHg (minus the offset), and *c* is the exponential rate constant.

Hypoxic responses were performed under control conditions and in the presence of pharmacological agents at concentrations consistent with those shown to modify mitochondrial succinate metabolism and mitoROS generation. These included diethyl Succinate [30] (DESucc, 1 and 5 mM, Sigma-Aldrich, Gillingham, UK), dimethyl malonate [19,30] (DMM, mitochondrial complex II inhibitor, Sigma-Aldrich, Gillingham, UK), MitoTEMPO [30,31] (20 µM, MitoT, mitochondrial antioxidant, targeted to the matrix, Sigma-Aldrich, Gillingham, UK), and 10-(6′-plastoquinonyl) decyltriphenylphosphonium [32,33] (1 µM, SKQ1, mitochondrial antioxidant targeted to the intermembrane space, Bio-Techne Ltd., Abingdon, UK). Incubation time was 5 min for DESucc and DMM and 20–25 min for MitoT and SKQ1 to allow for sufficient uptake.

### 2.3. Ventilatory Responses to Hypoxia and Hypercapnia

Respiratory parameters in unrestrained awake animals were recorded using whole body plethysmography (WBP) specifically designed for rats as described [34]. Gas flow into the chamber was approximately 2 L min^−1^. The WBP chamber was perfused with either a normoxic (78.97% N_2_, 21% O_2_, 0.03% CO_2_), hypoxic (89.97% N_2_, 10% O_2_, 0.03% CO_2_), or hypercapnic (73% N_2_, 21% O_2_, 6% CO_2_) gas mixture, controlled using Iox2.9.11.8 software (EMKA Technologies, Paris, France). Respiratory flow data were sampled at 1000 Hz and respiratory frequency (R_f_), tidal volume (V_T_), and minute ventilation (V_E_) were calculated offline.

WBP was performed between 8:30 a.m. to 14:00 p.m. Rats were individually placed in the WBP chamber and allowed to acclimatise for 15–30 min. Following acclimatisation, a 5-min baseline was recorded. Rats were then exposed to a cycle of hypoxia (10% O_2_, 5 min)/normoxia (5 min) and immediately after to a cycle of hypercapnia (6% CO_2_, 5 min)/normoxia (10 min). The final 2 min of hypoxic/hypercapnic exposure was used for analysis. Rats were removed from the WBP chamber and received an intraperitoneal (I.P.) injection of either vehicle or mitochondrial antioxidant (MitoT-1.96 μM kg^−1^/19.6 μM kg^−1^, SKQ1 500 nM kg^−1^). All solutions were prepared fresh on the day of experimentation and injection volume did not exceed 1 mL kg^−1^ bodymass. After one hour in the home cage, rats were placed back into the WBP chamber and the respiratory protocol was repeated.

### 2.4. Data Analysis

Data are presented as mean ± SEM or as box-whisker plots with median, mean (shown as +), the box representing the interquartile range and the whiskers extending to outliers. Single points represent individual chemoafferent fibres or animals. Statistical analysis was performed using (i) a paired 2-tailed student’s *t*-test, (ii) repeated measures one-way analysis of variance (ANOVA) or (iii) repeated measures two-way ANOVA with Tukey or Dunnett’s post hoc analysis where appropriate (Prism v9, GraphPad Software, San Diego, CA, USA). Significance was taken as *p* < 0.05.

## 3. Results

### 3.1. Succinate Causes Significant CB Chemoafferent Excitation

**Figure 1:**
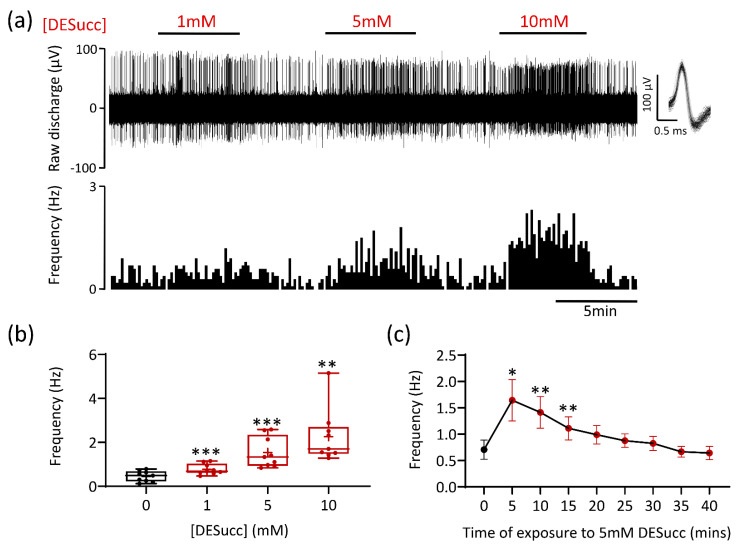
Succinate causes carotid body (CB) chemoafferent stimulation. (**a**) Example CB chemoafferent recording of the response to 1, 5, and 10 mM diethyl succinate (DESucc). Raw voltage is shown (upper) along with frequency histograms (lower). Overdrawn action potentials are inset, demonstrating single fibre discrimination. (**b**) Mean steady-state responses to DESucc at 1, 5, and 10 mM concentrations (*n* = 9 fibres, *N* = 4 animals). Data presented as box-whisker plots with median, mean (shown as +), the box representing the interquartile range and the whiskers extending to outliers. Single points represent individual fibres. ** and *** denote *p* < 0.01 and *p* < 0.001 vs. 0 mM, one-way repeated measures ANOVA with Dunnett’s post hoc test. (**c**) Time course of prolonged exposure to 5 mM DESucc (*n* = 11 fibres, *N* = 5 animals). Data presented as mean ± SEM. * and ** denote *p* < 0.05 and *p* < 0.01 vs. 0 min, one-way repeated measures ANOVA with Dunnett’s post hoc test.

**Figure 2:**
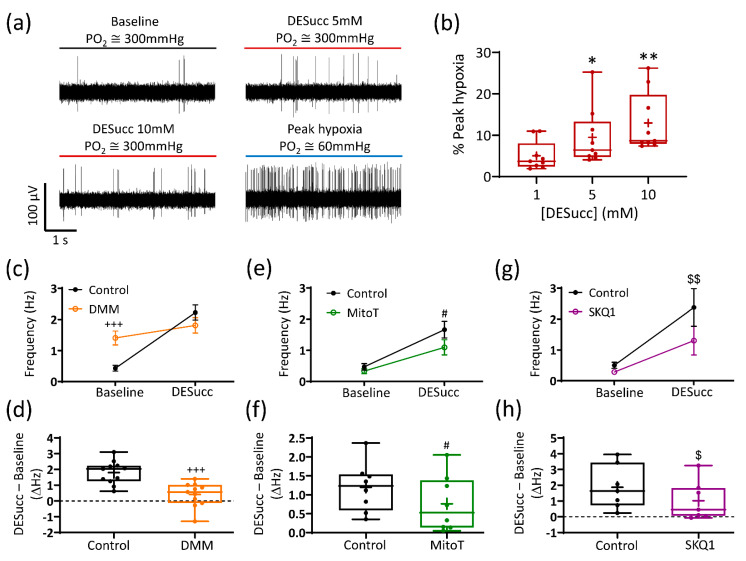
The carotid body (CB) response to succinate is dependent on mitochondrial complex II activity and reactive oxygen species generation. (**a**) Example 5 s raw CB chemoafferent recordings from the same fibre comparing activity in the presence of diethyl succinate (DESucc—5 and 10 mM) with peak hypoxia. (**b**) Mean responses to 1, 5, and 10 mM DESucc as a percentage of peak hypoxia (*n* = 9 fibres, *N* = 4 animals). * and ** denote *p* < 0.05 and *p* < 0.01 vs. 1 mM, one-way repeated measures ANOVA with Dunnett’s post hoc test. (**c**,**d**) Absolute and changes in frequency caused by 5 mM DESucc ± 10 mM dimethyl malonate (DMM), respectively (*n* = 11 fibres, *N* = 5 animals). (**e**,**f**) Absolute and changes in frequency caused by 5 mM DESucc ± 20 µM MitoTEMPO (MitoT), respectively (*n* = 8 fibres, *N* = 6 animals). (**g**,**h**) Absolute and changes in frequency caused by 5 mM DESucc ± 1 µM SKQ1, respectively (*n* = 6 fibres, *N* = 5 animals). For (**c**,**e**,**g**), data presented as mean ± SEM. +++ *p* < 0.001 control vs. DMM, # *p* < 0.05 control vs. MitoT, $$ *p* < 0.01 control vs. SKQ1, two-way ANOVA with Tukey post hoc test. For (**d**,**f**,**h**), data presented as box-whisker plots with median, mean (shown as +), the box representing the interquartile range and the whiskers extending to outliers. Single points represent individual fibres. +++ *p* < 0.001 control vs. DMM, # *p* < 0.05 control vs. MitoT, $ *p* < 0.05 control vs. SKQ1, paired *t*-test.

Application of the cell permeable compound diethyl succinate (DESucc; 1–10 mM) increased chemoafferent activity in a concentration dependent manner (Figure 1a,b). The onset of the response was quick, achieving steady state within 3 min, and was rapidly reversible (Figure 1a). At the highest concentration (10 mM), the frequency peaked within the range of 1.5–5 Hz (Figure 1b). In four experiments, application of 20 mM DESucc was tested but we did not observe any further rise in frequency, measuring 1.9 ± 0.7 Hz, *n* = 4. Prolonged exposure of the CB to 5 mM DESucc demonstrated that the chemoafferent frequency did not continue to rise, but rather peaked at 5 min, remained elevated at 15 min before returning to, or slightly below baseline (Figure 1c). Even at the highest concentrations, the response to DESucc was relatively modest compared to hypoxia (Figure 2a). Experiments performed on the same CB preparations showed that the maximum rise in chemoafferent frequency induced by DESucc was approximately 10–30% of that induced by a subsequent severe hypoxic stimulus (Figure 2a,b). Thus, although excessive succinate metabolism did cause CB stimulation, it did not precisely mimic hypoxia. Chemoafferent excitation induced by 5 mM DESucc was almost completely abolished by 10 mM DMM, a competitive inhibitor of mitochondrial complex II (Figure 2c,d). Furthermore, excitation caused by DESucc was attenuated in the presence of two different mitochondrial antioxidants MitoT (targeted to the mitochondrial matrix; 20 µM) and SKQ1 (targeted to the mitochondrial intermembrane space; 1 µM) (Figure 2e–h).

### 3.2. Excessive Succinate Metabolism Attenuates CB Hypoxic Sensitivity

**Figure 3:**
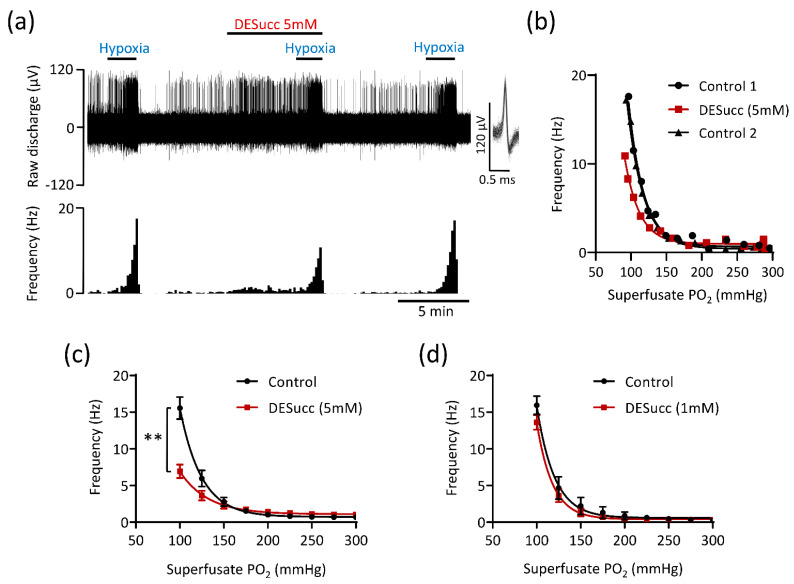
High levels of mitochondrial succinate metabolism depress carotid body hypoxic sensitivity. (**a**) Example CB chemoafferent recording of the response to hypoxia in the presence and absence of 5 mM diethyl succinate (DESucc). Raw voltage is shown (upper) along with frequency histograms (lower). Inset: overdrawn action potentials. (**b**) PO_2_-chemoafferent frequency response curves from a single experiment corresponding to the example shown in (**a**). (**c**) Mean chemoafferent hypoxic response curves for paired control and 5 mM DESucc (*n* = 9 fibres, *N* = 5 animals). (**d**) Mean chemoafferent hypoxic response curves for paired control and 1 mM DESucc (*n* = 5 fibres, *N* = 5 animals). Data presented as mean ± SEM. ** denotes *p* < 0.01 vs. control, two-way repeated measures ANOVA.

CB chemoafferent activity recorded during hypoxia exhibited a characteristic exponential increase below a certain PO_2_ threshold (Figure 3a,b). In the presence of 5 mM DESucc, chemoafferent frequency in hypoxia was significantly decreased and the PO_2_ ‘set-point’ for hypoxic response initiation was left-shifted, suggestive of an attenuation of O_2_ sensitivity (Figure 3a–c). The inhibition of the hypoxic response caused by DESucc was reversible (Figure 3a,b). Application of a lower concentration of DESucc (1 mM) did not alter the CB chemoafferent response to hypoxia (Figure 3d).

### 3.3. Mitochondrial Antioxidants and Inhibition of Succinate Metabolism Decrease but Do Not Abolish CB Chemoafferent Responses to Hypoxia

**Figure 4:**
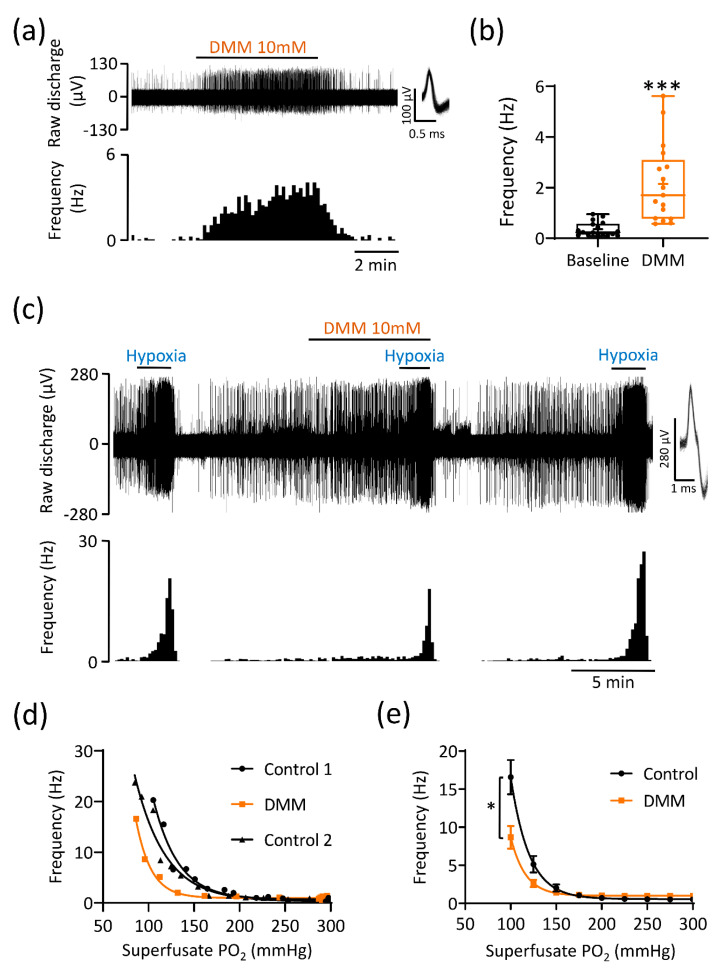
Inhibition of mitochondrial succinate metabolism causes basal carotid body (CB) chemoafferent excitation but partially attenuates the response to hypoxia. (**a**) Example chemoafferent response to 10 mM dimethyl malonate (DMM), an inhibitor of mitochondrial succinate dehydrogenase (complex II). Raw voltage (upper) is shown along with frequency histograms (lower). Inset: overdrawn action potentials. (**b**) Mean steady state frequency caused by DMM (*n* = 17 fibres, *N* = 10 animals). Data presented as box-whisker plots with median, mean (shown as +), the box representing the interquartile range and the whiskers extending to outliers. Single points represent individual fibres. *** denotes *p* < 0.001 vs. baseline, paired *t*-test. (**c**) Example chemoafferent response to hypoxia in the presence and absence of 10 mM DMM. (**d**) PO_2_-response curves from a single experiment corresponding to the example shown in (**c**). (**e**) Mean chemoafferent hypoxic response curves for paired control and 10 mM DMM (*n* = 8 fibres, *N* = 5 animals). Data presented as mean ± SEM. * denotes *p* < 0.05 vs. control, two-way repeated measures ANOVA.

To evaluate the importance of endogenous succinate metabolism, we monitored chemoafferent frequency in the presence of DMM (10 mM; a cell permeable and competitive inhibitor of SDH, complex II). In normoxia, DMM caused rapid and reversible chemostimulation in all preparations tested (Figure 4a,b). The elevation above baseline was variable, lying within the range of 0.5–5.5 Hz and was maintained throughout the stimulus duration (Figure 4a,b). DMM also significantly decreased the chemoafferent frequency in hypoxia and evoked a left shift in the hypoxic response curve (Figure 4c–e). However, a significant component (greater than 50%) of the overall hypoxic response was still preserved (Figure 4c–e). The inhibitory action of DMM on CB O_2_ sensitivity was reversible as evidenced by restoration of the response to hypoxia after 10–15 min washout (Figure 4c,d).

**Figure 5:**
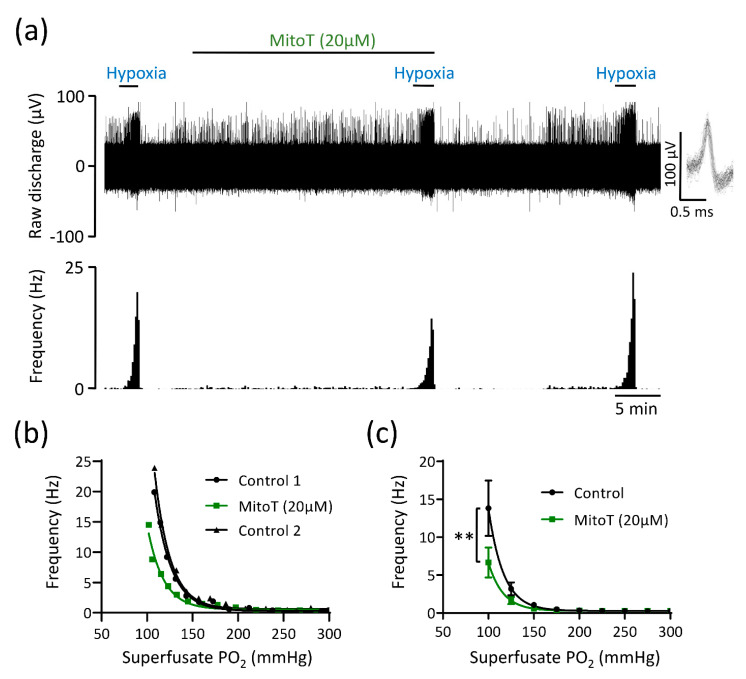
The mitochondrial antioxidant MitoTEMPO (MitoT) decreases but does not abolish carotid body (CB) chemoafferent responses to hypoxia. (**a**) Raw CB chemoafferent recording of the response to hypoxia ± 20 μM MitoT. Raw voltage is shown (upper) along with frequency histograms (lower). Inset: overdrawn action potentials from a single fibre. (**b**) PO_2_-chemoafferent frequency response curves from a single experiment corresponding to the example shown in (**a**). (**c**) Mean chemoafferent hypoxic response curves for paired control and 20 μM MitoT (*n* = 9 fibres, *N* = 6 animals). Data are presented as mean ± SEM. ** denotes *p* < 0.01 MitoT vs. control, two-way repeated measures ANOVA.

**Figure 6:**
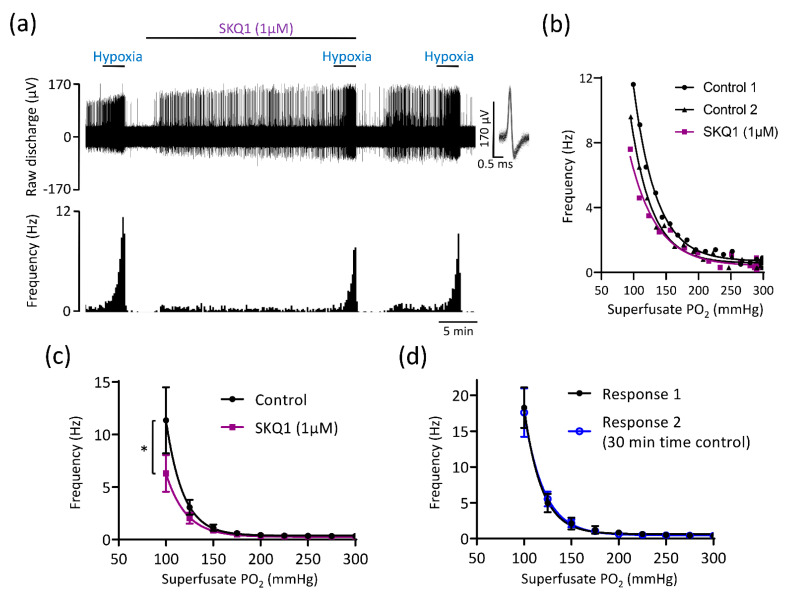
The mitochondrial antioxidant SKQ1 partially attenuates carotid body (CB) chemoafferent responses to hypoxia. (**a**) Raw CB chemoafferent recording of the response to hypoxia ± 1 μM SKQ1. Raw voltage is shown (upper) along with frequency histograms (lower). Inset: overdrawn action potentials from a single fibre. (**b**) PO_2_-chemoafferent frequency response curves from a single experiment corresponding to the example shown in (**a**). (**c**) Mean chemoafferent hypoxic response curves for paired control and 1 μM SKQ1 (*n* = 9 fibres, *N* = 6 animals). (**d**) Mean chemoafferent hypoxic response curves for 2 repeated control hypoxic exposures separated by 30 min (*n* = 7 fibres, *N* = 7 animals). For (**c**,**d**), data are presented as mean ± SEM. * denotes *p* < 0.05 SKQ1 vs. control, two-way repeated measures ANOVA.

As succinate increases mitoROS generation as a by-product of respiration [18,19,30], we assessed CB responses to hypoxia in presence of two different mitochondrial antioxidants MitoT (20 µM) and SKQ1 (1 µM). MitoT (which is targeted to the mitochondrial matrix) caused an attenuation in the rise in chemoafferent activity in hypoxia, without completely abolishing it, with a significant proportion of the response (>50%) being preserved (Figure 5a–c). MitoT induced a left shift in the PO_2_ threshold for response initiation, suggestive of a decrease in CB O_2_ sensitivity (Figure 5a–c). SKQ1, an antioxidant targeted to the mitochondrial intermembrane space, also decreased but did not fully eliminate the chemoafferent response to hypoxia (Figure 6a–c). At a superfusate PO_2_ of 100 mmHg, the response to hypoxia was blunted by approximately 20–50% (Figure 6a–c). SKQ1 also produced a left shift in the CB hypoxic response curve, signifying decreased O_2_ sensitivity (Figure 6a–c). Inhibition caused by MitoT and SKQ1 was not always reversible and there was a possibility that the decrease in O_2_ sensitivity was caused by a time dependent run-down during the 20–25 min incubation period. Additional experiments were performed comparing 2 control hypoxic responses 30 min apart. Paired chemoafferent responses for the same fibre separated by 30 min exhibited a high degree of consistency (Figure 6d), suggesting that the inhibition of CB O_2_ sensitivity observed in the presence of MitoT and SKQ1 was not due to time-dependent preparation run-down.

### 3.4. Mitochondrial Antioxidants Decrease Ventilatory Responses to Hypoxia but Not Hypercapnia

**Figure 7:**
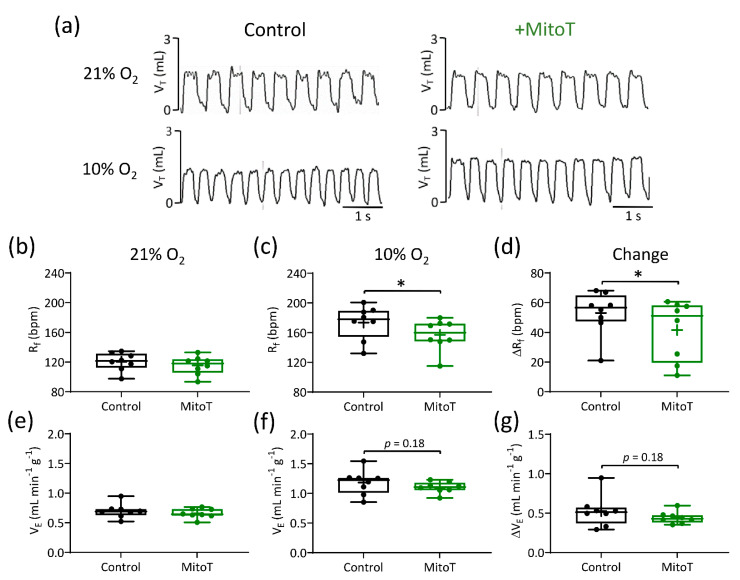
MitoTEMPO (MitoT) decreases the rise in respiratory frequency (R_f_) during hypoxia. (**a**) Representative respiratory traces (tidal volume (V_T_) vs. time, 5 s) illustrating breathing pattern before (**left**) and after (**right**) MitoT administration (19.6 µM kg^−1^, I.P.), in normoxia (21% O_2_) and during hypoxia (10% O_2_). (**b**–**d**) Mean R_f_ in normoxia, hypoxia and the relative change, respectively, before and after MitoT (*N* = 8 animals). (**e**–**g**) Mean minute ventilation (V_E_) in normoxia, hypoxia and the relative change, before and after MitoT. Data presented in box and whisker plots show mean (+), median line with a box range of 25th and 75th percentiles and outlier whiskers at minimum and maximum. Single points represent individual animals. * denotes *p* < 0.05, paired *t*-test.

Administration of a relatively low dose of MitoT (1.96 μM kg^−1^, I.P.) did not modify basal R_f_, V_T_, and V_E_ or the ventilatory response to hypoxia and hypercapnia (Table 1). At a higher dose (19.6 μM kg^−1^, I.P.), MitoT had no effect on normoxic R_f_ but did decrease the rise in R_f_ induced by hypoxia (Figure 7a–d, Table 1). The overall hypoxic ventilatory response (HVR) was unaffected, suggesting a partial compensation of increased V_T_ (Figure 7e–g, Table 1). The 19.6 μM kg^−1^, I.P. dose of MitoT had no impact on any component of the hypercapnic ventilatory response (Table 1).

**Figure 8:**
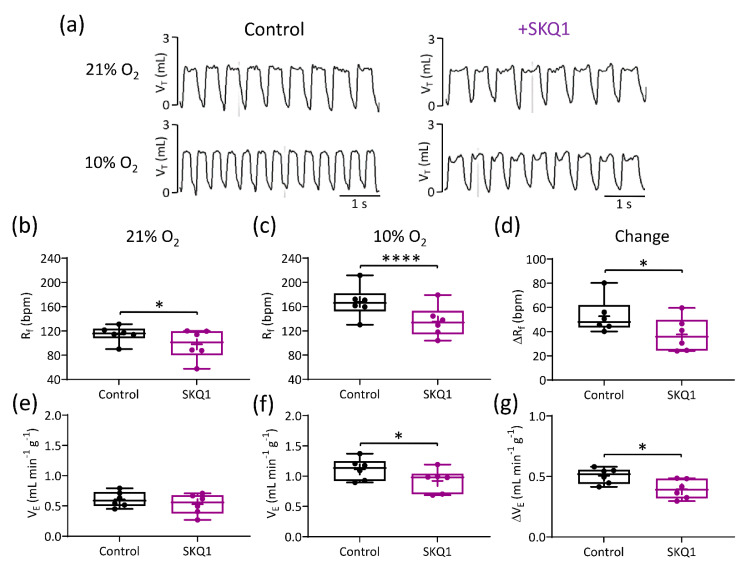
The mitochondrial antioxidant SKQ1 decreases the hypoxic ventilatory response (HVR). (**a**) Representative respiratory traces (tidal volume (V_T_) vs. time, 5 s) illustrating breathing pattern before (**left**) and after (**right**) SKQ1 administration (500 nM kg^−1^, I.P.), in normoxia (21% O_2_) and during hypoxia (10% O_2_). (**b**–**d**) Mean R_f_ in normoxia, hypoxia, and the relative change, respectively, before and after SKQ1 (*N* = 6 animals). (**e**–**g**) Mean minute ventilation (V_E_) in normoxia, hypoxia, and the relative change (HVR), before and after SKQ1. Data presented in box and whisker plots show mean (+), median line with a box range of 25th and 75th percentiles and outlier whiskers at minimum and maximum. Single points represent individual animals. * and **** denote *p* < 0.05 and *p* < 0.001 control vs. SKQ1 respectively, paired *t*-test.

Representative traces illustrating breathing pattern at baseline and in response to hypoxia in presence and absence of SKQ1 are presented in Figure 8a. Following SKQ1 administration (500 nM kg^−1^, I.P.), the basal R_f_ was markedly decreased, as was the elevation in R_f_ caused by hypoxia (Figure 8a–d, Table 1). Although there was still a robust increase in V_E_ during hypoxia, SKQ1 significantly attenuated the HVR by approximately 20% (Figure 8e–g). In contrast, SKQ1 had no effect on the response to hypercapnia (Table 1), suggesting that SKQ1 selectively inhibited hypoxic sensing within the whole animal.

Time/vehicle control experiments were performed before and 1 h after I.P. injection with vehicle (saline) and demonstrated consistent breathing patterns and responses to hypoxia and hypercapnia (Table 1).

## 4. Discussion

### 4.1. Main Findings

The present study shows that inhibition of succinate metabolism at mitochondrial complex II and mitochondrial antioxidants both partially attenuate the rat CB chemoafferent response to hypoxia. Mitochondrial antioxidants also decrease the rise in R_f_ in hypoxia and, in the case of SKQ1, the HVR. However, at the level of the CB and in the whole animal, a significant component (50–80%) of the response to hypoxia remains intact. Thus, whilst the data support a role for succinate metabolism and mitoROS being involved in CB and whole body O_2_ sensing, they are unlikely to be the sole mediators. Interestingly, excessive succinate metabolism causes modest chemoafferent stimulation in normoxia, but blunts activity in hypoxia. The additional inhibitory action of exaggerated succinate and mitoROS generation may have relevance in mediating CB dysfunction in disease.

### 4.2. Succinate and mitoROS Contribute to CB and Whole-Body Responses to Hypoxia in the Rat

Numerous studies have implicated the CB mitochondria in acute O_2_ sensing in multiple species [8,9,17,35]. CB mitochondrial cytochrome c oxidase has an unusually high K_m_ for O_2_ consistent with the PO_2_s known to cause type I cell stimulation [9]. Emerging evidence indicates that the low O_2_ affinity of CB mitochondria could be due to a unique expression profile of mitochondrial electron transport chain complex subunits [36,37] or the presence of a high level of a competitive inhibitor such as nitric oxide [35,38].

There is now much focus on identifying the precise link between mitochondrial inhibition and activation of the downstream chemotransduction cascade: K^+^ channel inhibition, membrane depolarisation, Ca^2+^ influx, neurotransmitter release, and chemoafferent excitation [35]. Our data identify an important role for succinate metabolism and mitoROS in mediating hypoxic sensitivity in the rat CB. Furthermore, we demonstrate that mitochondrial antioxidants and particularly SKQ1 can dampen the HVR in awake animals without impacting on hypercapnic ventilation. These findings are consistent with previous studies performed on isolated mouse type I cells where conditional deletion of mitochondrial complex I gene *ndufs2* prevented hypoxia induced mitochondrial intermembrane ROS generation and rises in intracellular Ca^2+^ [17,18]. Identifying a role for succinate and mitoROS in the intact CB preparation is an important finding as there are known differences between reduced and whole organ CB preparations in being able to sense and respond to other stimuli such as low glucose [39,40,41,42]. Furthermore, our data show that involvement of succinate and mitoROS in CB O_2_ sensing is conserved between species and is present in the rat, albeit to a lesser extent. Validation of a similar role in the human CB is still warranted.

### 4.3. Implications of the Current Study

In the presence of DMM, MitoT, and SKQ1, although aspects of the ex vivo CB and in vivo ventilatory response to hypoxia were depressed, they were far from abolished, with around 50–80% being preserved. The stimulation with exogenous succinate led to an increase in the basal nerve discharge frequency, however it did not mimic hypoxia. Excitation by succinate was completely abolished by DMM and was attenuated by MitoT and SKQ1. This is consistent with succinate causing chemostimulation via metabolism at complex II, reverse electron transport (RET), and mitoROS generation. It was hypothesised that co-stimulation of the CB with succinate and hypoxia would potentiate the hypoxic response as reported by Arias-Mayenco and colleagues (2018) in isolated mice type I cells. On the contrary, our data show that such co-stimulation leads to a depression of hypoxic responsiveness in the ex vivo nerve preparation. Therefore, excessive succinate metabolism and mitoROS generation have additional inhibitory actions on CB function that may be relevant in pathophysiology or cellular plasticity e.g., ageing.

Our data support the idea that physiological levels of mitoROS are important in mediating some of the hypoxic chemotransduction, but not all. Changes in MgATP, lactate, and possibly other, as yet unidentified substances, may be necessary for a full response to hypoxia to be evoked [13,15]. The specific role of each mediator could be dependent on the specific intensity of hypoxia as has been suggested for release of neurotransmitters/neuromodulators [43]. That said, there are questions over a role of lactate being involved in CB O_2_ sensing in the rat based on the recent finding that acute lactate administration (up to 20 mM) does not evoke type I cell depolarisation or chemoafferent excitation [44].

Alternatively, an upregulation of redundant control mechanisms in our experiments could account for the lack of complete abolition of the hypoxic response in the presence of DMM, MitoT, and SKQ1. The pharmacological approach used makes it difficult to separate out potential redundant control mechanisms, with those that are acting in parallel. If redundant mechanisms were at play in the current investigation, then they must have been induced very rapidly as pharmacological interventions were only applied for a maximum of 1 h. The lack of complete elimination of the response to hypoxia in the presence of DMM, MitoT, and SKQ1 also does not rule out the involvement of other mediators independent of mitochondrial function including H_2_S [45,46] and ROS derived from other sources such as NADPH oxidase [47,48]. Whilst mitoROS may be elevated in hypoxia, in other compartments, they may be decreased and the specific interactions between ROS and ion channels require further investigation. The importance of H_2_S has also been challenged by the findings that mice lacking cystathionine-γ-lyase (CSE) have preserved CB and ventilatory responses to hypoxia [49]. Again, O_2_ stimulus intensity is likely to account for some of these apparent discrepancies, with H_2_S generation now thought to be more relevant at moderate rather than severe hypoxic intensities [50]. Therefore, it is possible that there are many mediators, and their importance may be apparent at different severities of hypoxia, something that should be addressed in the future.

### 4.4. Translational Relevance

Lack of full understanding of the CB chemotransduction cascade undermines any attempts at designing potential treatment options for patients suffering from diseases associated with CB dysfunction, such as heart failure, hypertension, or obstructive sleep apnoea. One of the conclusions that can be drawn from the present study is that different levels of succinate metabolism, and consequently mitoROS, may have opposite effects on the CB activity and the response to hypoxia. The stimulatory impact of the succinate metabolism is likely to be important for initiation of the hypoxic response, whereas when very high excessive levels of succinate metabolism are reached, this may lead to detrimental accumulation of high levels of mitoROS. While ROS are known signalling molecules and are part of many signalling pathways, in severe excess, they cause damage to the cells and individual organelles, such as mitochondria. Providing that one of the hypotheses states that the oxygen sensor is located in the CB mitochondria, a major increase could have a detrimental impact on the overall oxygen sensing mechanism.

ROS have also been implicated in CB hyperactivity previously, although the focus so far has been on those derived from angiotensin II and NADPH oxidase [51,52,53,54,55,56]. Interestingly, it has been observed that mitochondrial superoxide dismutase nitration and protein expression is elevated in the CB following 7 days of chronic intermittent hypoxia (CIH), suggestive of elevated mitoROS production [57]. Our data do identify that mitochondrial selective antioxidants are capable of dampening CB function, which could offer a new approach to reducing CB hyperactivity in certain pathologies. Evaluating mitoROS generation and mitochondrial function in the CB in multiple pathologies will be an important next step.

In the current investigation, DMM inhibition of mitochondrial complex II resulted in a pronounced chemoafferent excitation. We speculate that basal stimulation by DMM was a consequence of a slight fall in intracellular MgATP, but this requires validation in future work. Accordingly, previous studies identified persistent type I cell membrane depolarisation in mice lacking one *SDHD* allele (SDHD^+/−^) [23]. These findings highlight a particularly high importance of succinate metabolism in maintaining normoxic electrical stability within the CB. Mutations in the *SDHD* gene are one of the causes of pheochromocytoma and paraganglioma, paragangliomas being the most prevalent type of CB cancer [58]. Whether or not the chronic stimulation itself contributes to oncogenesis (in addition to succinate dependent HIF1α stabilisation) is an area that has not yet been fully explored, particularly in combination with ageing.

### 4.5. Limitations

A major part of this study was performed on ex vivo nerve preparation rather than isolated type I cells. While chemoafferent recordings are a validated way of assessing the hypoxic sensitivity, it is impossible to determine whether the effects of antioxidants and inhibitors used in this study are limited to type I cells. There could be potential effects on other cell types present in the CB, such as type II cells or the nerve itself and therefore affect the final reading. Similar limitations apply to the in vivo studies as the antioxidants were administered systemically and they were not specifically targeted to the CB mitochondria. Therefore, some systemic effects on the cardiorespiratory system could have altered the response to hypoxia by disrupting ROS signalling. Finally, findings presented here were described in a rodent model, as such further studies are needed to determine whether the same effects are observed in humans.

## 5. Conclusions

Blocking succinate metabolism and the use of mitochondrial antioxidants decreases O_2_ sensing both at the level of the intact ex vivo CB and whole body HVR. However, significant proportions of the responses are preserved, suggesting that succinate-mediated mitoROS is not the only relevant signalling pathway. Excessive levels of succinate metabolism impair CB function in hypoxia. Treatment of the CB with mitochondrial selective antioxidants may offer a new approach for treating CB-related cardiovascular and respiratory disorders.

## Figures and Tables

**Table 1:** Changes in respiratory variables (R_f_—respiratory frequency; V_T_—tidal volume; V_E_—minute ventilation) in response to hypoxia or hypercapnia in the presence or absence of saline, MitoTEMPO or SKQ1.

Exposure	Intervention		N	ΔR_f_ (bpm)	ΔV_T_ (mL g^−1^)	ΔV_E_ (mL min^−1^ g^−1^)
Hypoxia (10% O_2_)	Vehicle control (saline)	−	5	42.2 ± 7	0.0012 ± 0.0004	0.39 ± 0.06
		+	5	43.7 ± 6	0.002 ± 0.0003	0.5 ± 0.02
	MitoTEMPO (1.96 μM kg^−1^)	−	6	57.8 ± 10.7	0.0012 ± 0.0002	0.47 ± 0.055
		+	6	50.6 ± 7.9	0.0014 ± 0.00025	0.4 ± 0.03
	MitoTEMPO (19.6 μM kg^−1^)	−	8	53 ± 5.3	0.001 ± 0.0002	0.53 ± 0.07
		+	8	41.6 ± 7.2 *	0.0015 ± 0.0004	0.44 ± 0.027
	SKQ1 (500 nM kg^−1^)	−	6	52.8 ± 6	0.0014 ± 0.00025	0.5 ± 0.03
		+	6	37.7 ± 5.7 *	0.0016 ± 0.0002	0.4 ± 0.03 *
Hypercapnia (6% CO_2_)	Vehicle control (saline)	−	5	81 ± 7	0.003 ± 0.0006	0.87 ± 0.14
		+	5	84.7 ± 11	0.003 ± 0.0005	0.86 ± 0.12
	MitoTEMPO (1.96 μM kg^−1^)	−	6	87 ± 8.5	0.003 ± 0.0003	0.94 ± 0.11
		+	6	80.9 ± 7.7	0.0018 ± 0.0002 *	0.75 ± 0.09
	MitoTEMPO (19.6 μM kg^−1^)	−	8	71.6 ± 5.5	0.003 ± 0.00025	0.98 ± 0.07
		+	8	66.7 ± 3.8	0.003 ± 0.0002	0.89 ± 0.05
	SKQ1 (500 nM kg^−1^)	−	6	68.6 ± 3.8	0.0025 ± 0.0005	0.78 ± 0.06
		+	6	72.6 ± 8	0.003 ± 0.0005	0.82 ± 0.07

* *p* < 0.05; paired *t*-test. Vehicle control—*n* = 5; MitoTEMPO (1.96 μM kg^−1^)—*n* = 6; MitoTEMPO (19.6 μM kg^−1^)—*n* = 8; SKQ1(500 nM kg^−1^)—*n*= 6.

## Data Availability

Throughout the manuscript, individual data points are presented and indicate averaged data from a single animal. Therefore, data generated or analysed during this study are included in this published article. The data presented in this study are available on request from the corresponding author.
